# JSH practical guidelines for hematological malignancies, 2023: II. lymphoma 9—adult T-cell leukemia–lymphoma (ATL)

**DOI:** 10.1007/s12185-025-04011-2

**Published:** 2025-06-04

**Authors:** Kisato Nosaka, Takuya Fukushima

**Affiliations:** 1https://ror.org/02vgs9327grid.411152.20000 0004 0407 1295Cancer Center, Kumamoto University Hospital Cancer Center, 1-1-1 Honjo, Chuo-Ku, Kumamoto, 860-8556 Japan; 2https://ror.org/02z1n9q24grid.267625.20000 0001 0685 5104School of Health Sciences, Faculty of Medicine & Graduate School of Health Sciences, Hematology and Immunology, University of the RyuKyus, Nakagami-Gun, Okinawa, Japan

**Keywords:** ATL, HTLV-1, Allogeneic HSCT

## Overview

Adult T-cell leukemia–lymphoma (ATL) was proposed as a disease concept describing T-cell neoplasms caused by human T-cell leukemia virus type 1 (HTLV-1) infection and is common in southwestern Japan, especially the Kyushu region.^1–5^ In the 2017 WHO classification, ATL is classified as a mature T-cell neoplasm.^6^


Approximately 10 to 20 million people worldwide are infected with HTLV-1, with endemic areas including Japan, Africa, Central and South America, the Caribbean, Australia, and Melanesia. The number of HTLV-1 carriers in Japan is reported to be 1.08 million, but currently appears to be decreasing further.^7^

The lifetime incidence of ATL in HTLV-1 carriers is thought to be 2–5%, or nearly 1000 people per year. Recent nationwide surveys in Japan showed an average age of onset at 68 years, older than the average reported in surveys from the 1980s.^7,8^ ATL presents as leukocytosis with proliferation of abnormal lymphocytes called flower cells due to their characteristic petal-shaped nuclei, along with a variety of symptoms including lymphadenopathy, hepatosplenomegaly, skin rash, elevated blood LDH, hypercalcemia, and opportunistic infections.

Shimoyama et al. proposed four clinical subtypes (acute, lymphoma, chronic, and smoldering) based on a nationwide survey in 813 patients with ATL conducted by the Japan Clinical Oncology Group (JCOG) Lymphoma Study Group (LSG) in 1991 (Table 1).^9^ According to a nationwide clinico-epidemiological survey in Japan of ATL diagnosed between 2012 and 2013, relative frequencies were 51.9% for acute, 24.9% for lymphoma, 12.5% for chronic, and 10.7% for smoldering.^10^ Among these subtypes, acute and lymphoma ATL and chronic ATL with unfavorable prognostic factors (abnormal level of at least one laboratory value among LDH, albumin, and BUN) are known to often take a rapid course, and are collectively referred to as aggressive ATL. In contrast, chronic ATL without unfavorable prognostic factors and smoldering ATL take a relatively slow course, and are called indolent ATL. This classification and these categories are still widely used for diagnosis and treatment today.

Regarding prognosis, a nationwide survey in 770 patients diagnosed with ATL between 2010 and 2011 showed that 4-year overall survival (OS) rates were 16.8% for acute ATL, 19.6% for lymphoma ATL, 26.6% for chronic ATL with unfavorable prognostic factors, 62.1% for chronic ATL without unfavorable prognostic factors, and 59.8% for smoldering ATL.^11^

A retrospective study of 807 patients with acute or lymphoma ATL diagnosed between 2000 and 2009 at 81 institutions across Japan identified clinical stage, performance status, age, albumin, and soluble interleukin 2 receptor (sIL-2R) as prognostic factors. Patients were divided into three risk groups by number of prognostic factors, and median survival time (MST) was 16.2, 7.3, and 3.6 months in the low-, intermediate-, and high-risk groups, respectively.^12^ In addition, JCOG–LSG has continued to conduct clinical trials of aggressive ATL, which is why the JCOG response assessment criteria have come to be widely used in evaluation of responsiveness to chemotherapy (Table 2). In a pooled analysis of 276 patients from three clinical trials by JCOG–LSG, patients were divided into two groups based on performance status and hypercalcemia, and MST in these groups was 6.3 months and 17.8 months, respectively.^13^ Furthermore, a retrospective analysis of 1792 patients with acute or lymphoma ATL (2000–2013) aged 70 years or younger identified subtype (acute), performance status, hypercalcemia, C-reactive protein, and sIL-2R as prognostic factors. When patients were classified into three risk groups by number of prognostic factors, MST was 626, 322, and 197 days in the low-, intermediate-, and high-risk groups, respectively.^14^
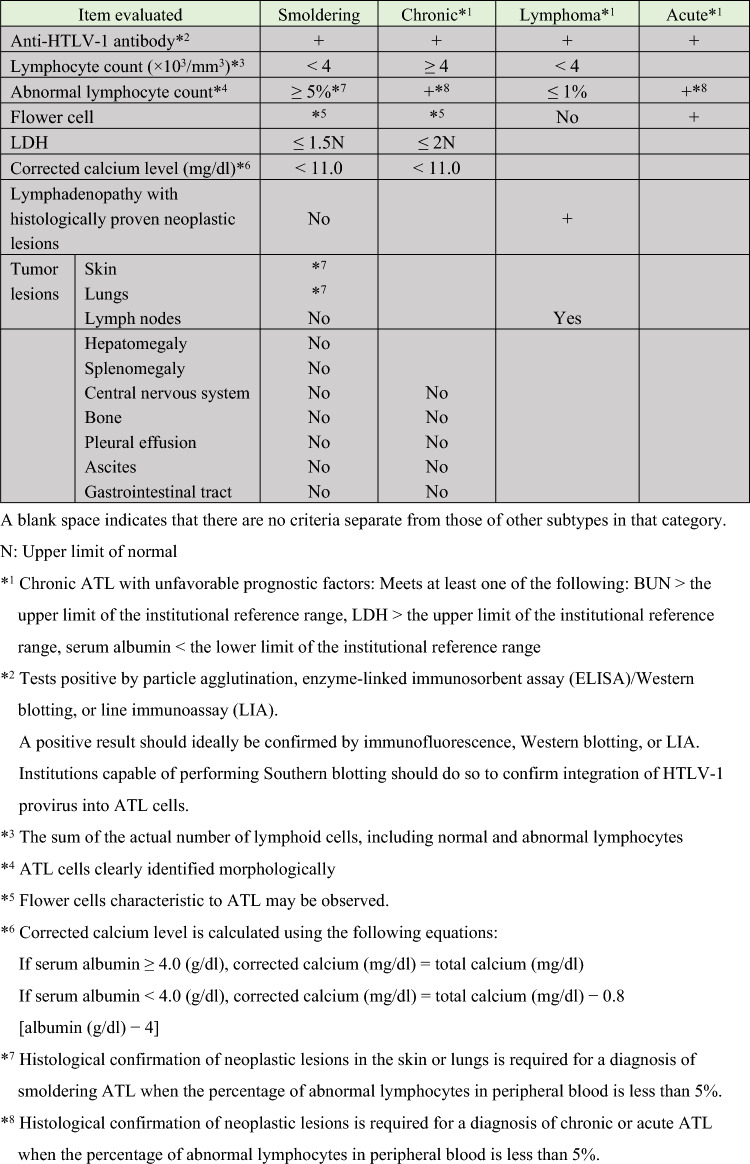


(Modified from Reference 9).
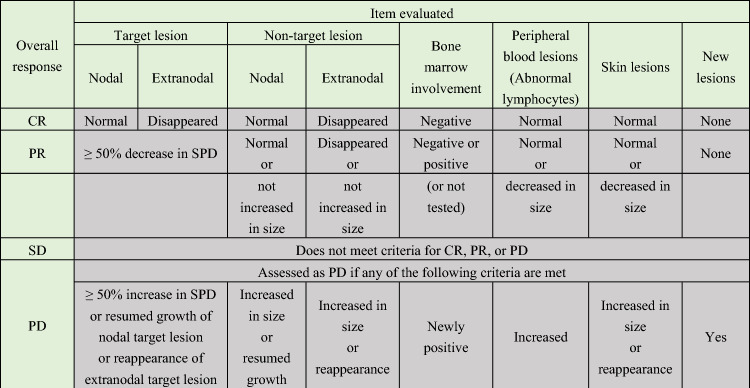


The overall assessment is not evaluable (NE) if any of the above items are not evaluable.

If target lesion is not present at baseline.
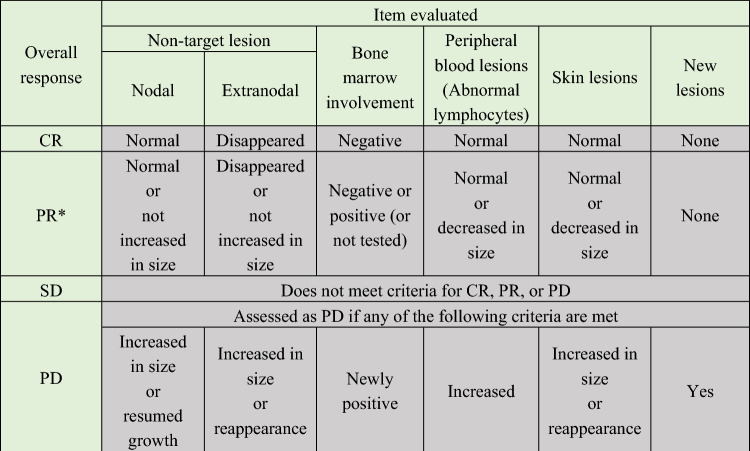


## References

1)Uchiyama T, et al. Adult T-cell leukemia: clinical and hematologic features of 16 cases. Blood. 1977; 50(3): 481–92.

2)Poiesz BJ, et al. Detection and isolation of type C retrovirus particles from fresh and cultured lymphocytes of a patient with cutaneous T-cell lymphoma. Proc Natl Acad Sci U S A. 1980; 77(12): 7415–9.

3)Hinuma Y, et al. Adult T-cell leukemia: antigen in an ATL cell line and detection of antibodies to the antigen in human sera. Proc Natl Acad Sci U S A. 1981; 78(10): 6476–80.

4)Yoshida M, et al. Monoclonal integration of human T-cell leukemia provirus in all primary tumors of adult T-cell leukemia suggests causative role of human T-cell leukemia virus in the disease. Proc Natl Acad Sci U S A. 1984; 81(8): 2534–7.

5)Miyoshi I, et al. Type C virus particles in a cord T-cell line derived by co-cultivating normal human cord leukocytes and human leukaemic T-cells. Nature. 1981; 294(5843): 770–1.

6)Ohshima K, et al. Adult T-cell leukemia/lymphoma. Swerdlow SH, et al. eds. WHO Classification of Tumours of Haematopoietic and Lymphoid Tissues. Lyon. IARC; 2017: pp363–7.

7)Iwanaga M. Epidemiology of HTLV-1 Infection and ATL in Japan: An Update. Front Microbiol. 2020; 11: 1124.

8)Nosaka K, et al. Epidemiological and clinical features of adult T-cell leukemia-lymphoma in Japan, 2010–2011: A nationwide survey. Cancer Sci. 2017; 108(12): 2478–86.

9)Shimoyama M, et al. Diagnostic criteria and classification clinical subtypes of adult T-cell leukemia-lymphoma. Br J Haematol. 1991; 79(3): 428-37.

10)Ito S, et al. Epidemiology of adult T-cell leukemia-lymphoma in Japan: An updated analysis, 2012-2013. Cancer Sci. 2021; 112(10): 4346–54.

11)Imaizumi Y, et al. Prognosis of patients with adult T-cell leukemia/lymphoma in Japan: A nationwide hospital-based study. Cancer Sci. 2020; 111(12): 4567–80. (3iiiA)

12)Katsuya H, et al. Prognostic index for acute- and lymphoma-type adult T-cell leukemia/lymphoma. J Clin Oncol. 2012; 30(14): 1635–40.

13)Fukushima T, et al. Japan Clinical Oncology Group (JCOG) prognostic index and characterization of long-term survivors of aggressive adult T-cell leukaemia-lymphoma (JCOG0902A). Br J Haematol. 2014; 166(5): 739–48.

14)Fuji S, et al. Development of a modified prognostic index for patients with aggressive adult T-cell leukemia-lymphoma aged 70 years or younger: possible risk-adapted management strategies including allogeneic transplantation. Haematologica. 2017; 102(7): 1258–65. (3iiiA)

## Algorithm



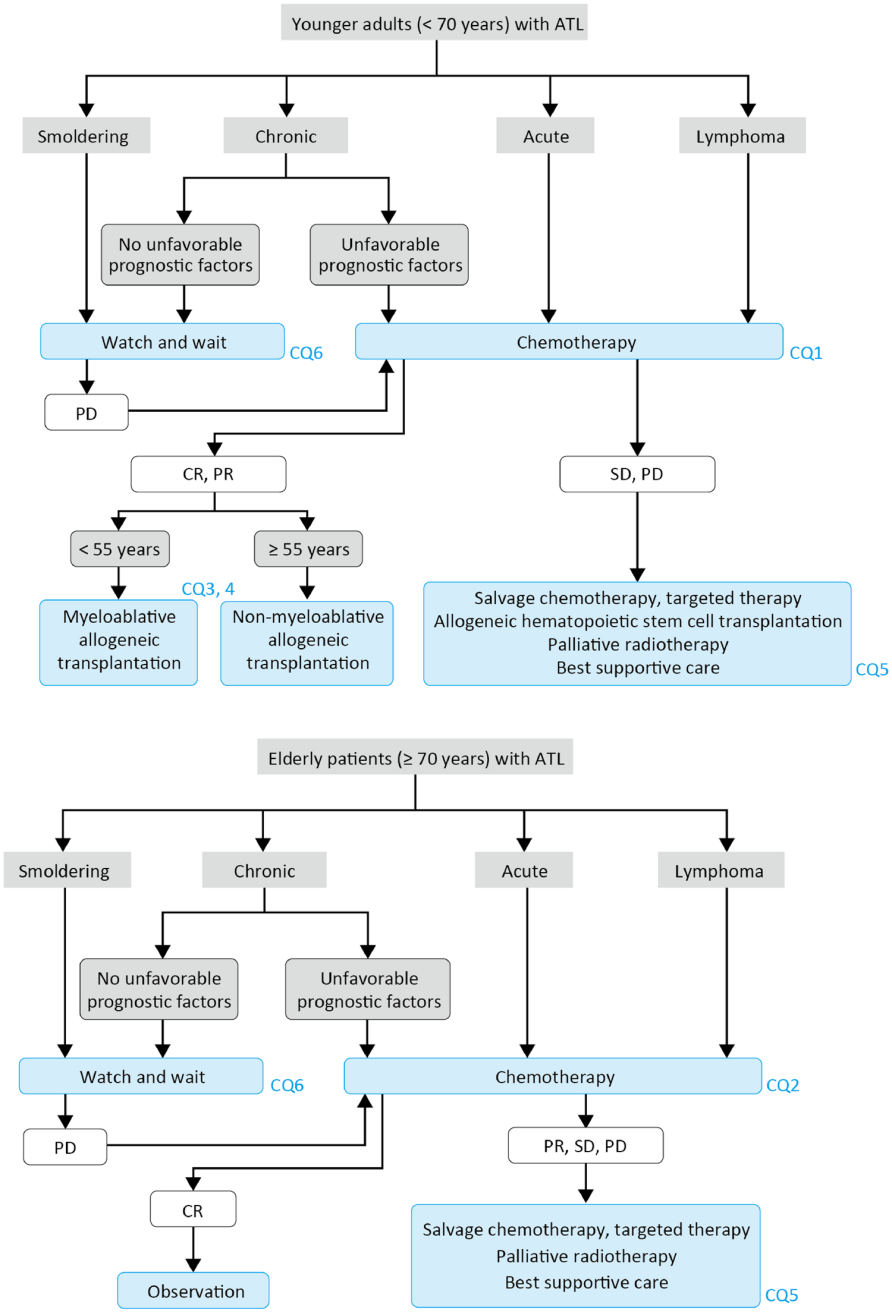


Among ATL subtypes, acute, lymphoma and chronic ATL with unfavorable prognostic factors (among high LDH, low albumin, and high BUN) are referred to as aggressive ATL, and should be treated with combination chemotherapy (CQ1, 2). Because allogeneic hematopoietic stem cell transplantation (HSCT) is generally performed in patients up to 70 years, this algorithm divides patients into two age groups: younger than 70 years and 70 years or older. Allogeneic HSCT should be considered for patients younger than 70 years who respond to chemotherapy and have suitable performance status and major organ function (CQ3). HSCT from an HLA-matched related or unrelated donor is the first choice, but cord blood transplantation or HLA-haploidentical HSCT may also be considered if a suitable donor is not available (CQ4). For relapsed or refractory ATL, chemotherapy or molecular targeted agents may be considered (CQ5). Indolent ATL, namely, chronic ATL without unfavorable prognostic factors and smoldering ATL, should be observed without treatment until progression to aggressive ATL, because the benefit of any form of early intervention remains unclear (CQ6).


**CQ1 What is the recommended treatment for younger adults (< 70 years) with newly diagnosed aggressive ATL?**

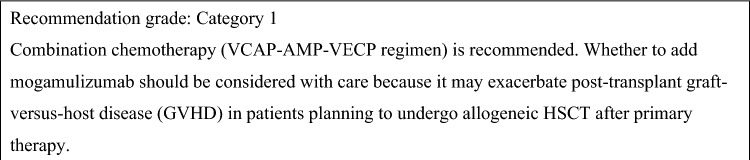



## Explanation

Various regimens for ATL have been investigated in clinical trials since the 1980s, but the prognosis was extremely poor, with a mean survival time of less than 1 year.^1^ Later, JCOG–LSG proposed a clinical classification system for ATL, and continued to conduct clinical trials of aggressive ATL, the subtypes for which treatment is indicated.^2,3^ An regimen intensified with eight cytotoxic anticancer drugs and granulocyte colony-stimulating factor and including intrathecal injection of methotrexate and prednisolone (LSG15) demonstrated better outcomes than those for previous treatments.^4^ In addition, a randomized controlled phase III trial compared VCAP–AMP–VECP (VCAP: vincristine, cyclophosphamide, doxorubicin, and prednisolone; AMP: doxorubicin, ranimustine, and prednisolone; VECP: vindesine, etoposide, carboplatin, and prednisolone), a modified version of LSG15 with fewer cycles plus intrathecal cytarabine, against CHOP-14 (cyclophosphamide, doxorubicin, vincristine, and prednisolone), a regimen used for non-Hodgkin lymphoma. The trial showed that VCAP–AMP–VECP was superior to CHOP-14 in terms of both complete response (CR) rate and OS, despite high hematologic toxicity,^5^ and established VCAP–AMP–VECP as a standard therapy.

A randomized controlled phase II trial investigated VCAP–AMP–VECP in combination with mogamulizumab, an anti-CC chemokine receptor 4 (CCR4) antibody targeting the chemokine receptor CCR4 that is expressed on more than 90% of ATL cells,^6^ and found that this combination was superior to VCAP–AMP–VECP alone in terms of CR rate.^7,8^ Based on these results, the indication for mogamulizumab was expanded to include patients with newly diagnosed CCR4-positive ATL. However, addition of mogamulizumab produced no significant differences in OS or progression-free survival (PFS). In addition, use of mogamulizumab before allogeneic HSCT should be considered carefully, because increased frequency of GVHD, severe disease, and transplant-related mortality (TRM) has been reported with use of mogamulizumab before allogeneic HSCT.^9^

Based on only one randomized controlled phase III trial in newly diagnosed aggressive ATL with results available at this time, VCAP–AMP–VECP therapy is recommended. However, VCAP–AMP–VECP is only indicated for patients younger than 70 years, based on the age range in that trial and the results of a subsequent retrospective analysis.^10^

Brentuximab vedotin, an antibody–drug conjugate composed of monomethyl auristatin E and anti-CD30 antibody, demonstrated benefit in a trial that compared BV–CHP (brentuximab vedotin, cyclophosphamide, doxorubicin, and prednisolone) against CHOP in previously untreated CD30-positive peripheral T-cell lymphoma,^11^ and is covered by the Japanese National Health Insurance (NHI). Brentuximab vedotin can be used for CD30-positive, newly diagnosed aggressive ATL, but further efficacy evaluation is needed, because only a small number of ATL patients were enrolled in the trial.

## References

1) Shimoyama M, et al. Chemotherapeutic results and prognostic factors of patients with advanced non-Hodgkin’s lymphoma treated with VEPA or VEPA-M. J Clin Oncol. 1988; 6(1): 128–41. (1iiDiv)

Shimoyama M, et al. Major prognostic factors of adult patients with advanced T-cell lymphoma/leukemia. J Clin Oncol 1988; 6(7): 1088-97. (2A)

2)Tsukasaki K, et al. Deoxycoformycin-containing combination chemotherapy for adult T-cell leukemia-lymphoma: Japan Clinical Oncology Group Study (JCOG9109). Int J Hematol. 2003; 77(2): 164–70. (3iiiDiv)

3)Yamada Y, et al. A new G-CSF-supported combination chemotherapy, LSG15, for adult T-cell leukaemia-lymphoma: Japan Clinical Oncology Group Study 9303. Br J Haematol. 2001; 113(2): 375–82. (3iiiA)

4)Tsukasaki K, et al. VCAP-AMP-VECP compared with biweekly CHOP for adult T-cell leukemia-lymphoma: Japan Clinical Oncology Group Study JCOG9801. J Clin Oncol. 2007; 25(34): 5458–64. (1iiA)

5)Ishida T, et al. Clinical significance of CCR4 expression in adult T-cell leukemia/lymphoma: its close association: with skin involvement and unfavorable outcome. Clin Cancer Res. 2003; 9(10Pt1): 3625–34. (3iiiA)

6)Ishida T, et al. Dose-intensified chemotherapy alone or in combination with mogamulizumab in newly diagnosed aggressive adult T-cell leukaemia-lymphoma: a randomized phase II study. Br J Haematol. 2015; 169(5): 672–82. (3iiiDiv)

7)Ishida T, et al. Follow-up of a randomised phase II study of chemotherapy alone or in combination with mogamulizumab in newly diagnosed aggressive adult T-cell leukaemia-lymphoma: impact on allogeneic haematopoietic stem cell transplantation. Br J Haematol. 2019; 184(3): 479–83. (3iiiDiv)

8)Fuji S, et al. Pretransplantation Anti-CCR4 Antibody Mogamulizumab Against Adult T-Cell Leukemia/Lymphoma Is Associated With Significantly Increased Risks of Sever and Corticosteroid-Refractory Graft-Versus-Host Disease, Nonrelapse Mortality, and Overall Mortality. J Clin Oncol. 2016; 34(28): 3426–33. (3iiiA)

9)Fuji S, et al. VCAP-AMP-VECP as a preferable induction chemotherapy in transplant-eligible patients with aggressive adult T-cell leukemia-lymphoma: a propensity score analysis. Bone Marrow Transplant. 2019; 54(9): 1399–405. (3iiiA)

10)Horwitz S, et al. Brentuximab vedotin with chemotherapy for CD30-positive peripheral T-cell lymphoma (ECHELON-2): a global, double-blind, randomised, phase 3 trial. Lancet. 2019; 393(10168): 229–40. (1iDiii)


**CQ2 What treatments are recommended for elderly patients (≥ 70 years) with newly diagnosed aggressive ATL?**

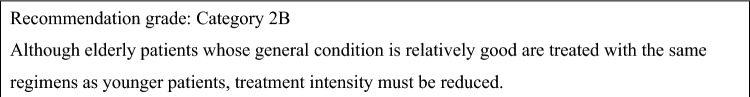



## Explanation

No prospective trial has investigated treatment of newly diagnosed aggressive ATL exclusively in elderly patients. The JCOG9801 trial, the only randomized phase III trial in aggressive ATL, established VCAP–AMP–VECP as the standard of care, but the trial only included patients younger than 70 years, and a subgroup analysis showed no significant difference in OS between VCAP–AMP–VECP and CHOP-14 in patients aged 56 years and older.^1^ A single-center, retrospective analysis in 34 patients with aggressive ATL aged 70 years or older showed that reduced-dose VCAP–AMP–VECP yielded MST of 13.4 months, making it a treatment option for elderly patients.^2^ In a randomized phase II trial of VCAP–AMP–VECP plus mogamulizumab vs. VCAP–AMP–VECP alone that included patients aged 70 years and older, combination therapy was superior in terms of CR rate, but had a higher rate of adverse events.^3^ A follow-up study showed no difference in OS and PFS between groups, and thus no conclusion was reached regarding the additive effect of mogamulizumab.^4^

Patients aged 70 years and older were also included in trials of the THP–COP regimen (cyclophosphamide, pirarubicin, vincristine, and prednisolone)^5^ and modified EPOCH regimen (etoposide, doxorubicin, cyclophosphamide, vincristine, and prednisolone).^6^ Both regimens were shown to be options, but most patients required dose reduction. In a Japanese nationwide survey on the prognosis of aggressive ATL diagnosed between 2010 and 2011, 145 of the total of 770 patients had acute ATL, were 70 years or older, and received chemotherapy, and CHOP-like therapy was the most common treatment option in that group followed by VCAP–AMP–VECP-like therapy.^7^

Elderly patients with aggressive ATL can be expected to respond to the same regimens used in younger patients, but treatment intensity should be reduced in consideration of age and comorbidities.

## References

1)Tsukasaki K, et al. VCAP-AMP-VECP compared with biweekly CHOP for adult T-cell leukemia-lymphoma: Japan Clinical Oncology Group Study JCOG9801. J Clin Oncol. 2007; 25(34): 5458–64. (1iiA)

2)Makiyama J, et al. Treatment outcome of elderly patients with aggressive adult T cell leukemia-lymphoma: Nagasaki University Hospital experience. Int J Hematol. 2014; 100(5): 464–72. (3iiA)

3)Ishida T, et al. Dose-intensified chemotherapy alone or in combination with mogamulizumab in newly diagnosed aggressive adult T-cell leukaemia-lymphoma: a randomized phase II study. Br J Haematol. 2015; 169(5): 672–82. (3iiiDiv)

4)Ishida T, et al. Follow-up of a randomised phase II study of chemotherapy alone or in combination with mogamulizumab in newly diagnosed aggressive adult T-cell leukaemia-lymphoma: impact on allogeneic haematopoietic stem cell transplantation. Br J Haematol. 2019; 184(3): 479–83. (3iiiDiii)

5)Takamatsu Y, et al. THP-COP regimen for the treatment of peripheral T-cell lymphoma and adult T-cell leukemia/lymphoma: a multicenter phase II study. Eur J Haematol. 2010; 84(5): 391–7. (3iiiDiv)

6)Tsukamoto Y, et al. Efficacy and Safety of the Modified EPOCH Regimen (Etoposide, Vincristine, Doxorubicin, Carboplatin, and Prednisolone) for Adult T-cell Leukemia/Lymphoma: A Multicenter Retrospective Study. Clin Lymphoma Myeloma Leuk. 2020; 20(7): e445–53. (3iiiDiv)

7)Imaizumi Y, et al. Prognosis of patients with adult T-cell leukemia/lymphoma in Japan: A nationwide hospital-based study. Cancer Sci. 2020; 111(12): 4567–80. (3iiiA)


**CQ3 Is allogeneic HSCT recommended for aggressive ATL?**

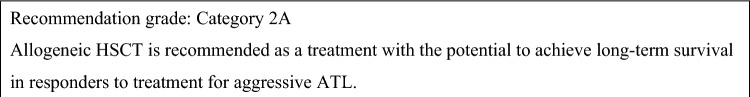



## Explanation

Allogeneic HSCT for aggressive ATL has been widely practiced since the 1990s, and promising results have been reported.^1–4^ A large retrospective study using a Japanese database showed that the 3-year OS rate in 386 ATL patients who underwent allogeneic HSCT was 33%.^5^ A prospective observational study showed similar outcomes.^6^ A Japanese nationwide survey on the prognosis of ATL diagnosed between 2010 and 2011 showed that allogeneic HSCT produced more promising outcomes than chemotherapy alone,^7^ and even eliminated ATL clones that persisted after chemotherapy in some patients,^8^ though it should be noted that only a limited number of patients in the survey had received allogeneic HSCT. On the basis of this evidence, allogeneic HSCT is recommended in responders to prior treatment, despite its high TRM rate.^9,10^ Immunological mechanisms mediated by donor-derived immunocompetent cells appear to be involved in response to allogeneic HSCT.^11^

Large retrospective analyses of conditioning regimens for allogeneic HSCT have shown no difference in OS or relapse rate between myeloablative and non-myeloablative conditioning.^12–16^ The upper age limit for myeloablative conditioning is currently 55 years, and non-myeloablative conditioning is typically performed for patients aged 50–70 years.

An HLA-matched related or unrelated donor is the first choice for donor selection.^5^ Although the previously mentioned large retrospective analysis identified cord blood as an unfavorable prognostic factor,^5^ results of more recent retrospective analyses show that use of cord blood is feasible.^6,17,18^ However, use of cord blood should be considered carefully, because it does not yield superior outcomes compared to using an HLA-matched related or unrelated donor. A case of allogeneic transplantation from HTLV-1 carrier blood donor that relapsed due to donor HTLV-1-infected cells has been reported.^19^ Consequently, when an HTLV-1 carrier is the donor, it is recommended to confirm the absence of clinical ATL (e.g., lymphadenopathy and abnormal lymphocytosis) in the donor. It is also advisable to confirm the absence of monoclonal or oligoclonal HTLV-1-infected cells by Southern blot analysis for HTLV-1 in peripheral blood, although this test is not covered by the Japanese NHI.^20^

Because treatment with mogamulizumab prior to allogeneic HSCT has been shown to increase TRM and worsen transplant outcomes, the use of mogamulizumab should be carefully considered in patients who may undergo transplantation.^21^

## References

1)Utsunomiya A, et al. Improved outcome of adult T-cell leukemia/lymphoma with allogeneic hematopoietic stem cell transplantation. Bone Marrow Transplant. 2001; 27(1): 15–20. (3iiiA)

2)Kami M, et al. Allogeneic haematopoietic stem cell transplantation for the treatment of adult T-cell leukemia/ lymphoma. Br J Haematol. 2003; 120(2): 304–9. (3iiiA)

3)Kato K, et al. Allogeneic bone marrow transplantation from unrelated human T-cell leukemia virus-I-negative donors for adult T-cell leukemia/lymphoma: retrospective analysis of data from the Japan Marrow Donor Program. Biol Blood Marrow Transplant. 2007; 13(1): 90–9. (3iiiA)

4)Fukushima T, et al. Allogeneic hematopoietic stem cell transplantation provides sustained long-term survival for patients with adult T-cell leukemia/lymphoma. Leukemia. 2005; 19(5): 829–34. (3iiiA)

5)Hishizawa M, et al. Transplantation of allogeneic hematopoietic stem cells for adult T-cell leukemia: a nationwide retrospective study. Blood. 2010; 116(8): 1369–76. (3iiiA)

6)Ito A, et al. Improved survival of patients with aggressive ATL by increased use of allo-HCT: a prospective observational study. Blood Adv. 2021; 5(20): 4156–66. (3iiiA)

7)Imaizumi Y, et al. Prognosis of patients with adult T-cell leukemia/lymphoma in Japan: A nationwide hospital-based study. Cancer Sci. 2020; 111(12): 4567–80. (3iiiA)

8)Yamasaki R, et al. Small number of HTLV-1-positive cells frequently remains during complete remission after allogeneic hematopoietic stem cell transplantation that are heterogeneous in origin among cases with adult T-cell leukemia/lymphoma. Leukemia. 2007; 21(6): 1212–7.

9)Inoue Y, et al. Prognostic importance of pretransplant disease status for posttransplant outcomes in patients with adult T cell leukemia/lymphoma. Bone Marrow Transplant. 2018; 53(9): 1105–15. (3iiiA)

10)Fuji S, et al. Early application of related SCT might improve clinical outcome in adult T-cell leukemia/lymphoma. Bone Marrow Transplant. 2016; 51(2): 205–11. (3iiiA)

11)Kanda J, et al. Impact of graft-versus-host disease on outcome after allogeneic hematopoietic cell transplantation for adult T-cell leukemia: a retrospective cohort study. Blood. 2012; 119(9): 2141–8. (3iiiA)

12)Ishida T, et al. Allogeneic hematopoietic stem cell transplantation for adult T-cell leukemia-lymphoma with special emphasis on preconditioning regimen: a nationwide retrospective study. Blood. 2012; 120(8): 1734–41. (3iiiA)

13)Okamura J, et al. Allogeneic stem-cell transplantation with reduced conditioning intensity as a novel immunotherapy and antiviral therapy for adult T-cell leukemia/lymphoma. Blood. 2005; 105(10): 4143–5. (2A)

14)Tanosaki R, et al. Allogeneic hematopoietic stem cell transplantation using reduced-intensity conditioning for adult T-cell leukemia/lymphoma: impact of antithymocyte globulin on clinical outcome. Biol Blood Marrow Transplant. 2008; 14(6): 702–8. (3iiiA)

15)Choi I, et al. Long-term outcome after hematopoietic SCT for adult T-cell leukemia/lymphoma: results of prospective trials. Bone Marrow Transplant. 2011; 46(1): 116–8. (3iiiA)

16)Inoue Y, et al. Impact of conditioning intensity and regimen on transplant outcomes in patients with adult T-cell leukemia-lymphoma. Bone Marrow Transplant. 2021; 56(12): 2964–74. (3iiiA)

17)Muranushi H, et al. GVHD-free, relapse-free survival provides novel clues for optimizing allogeneic-HSCT for adult T-cell leukemia/lymphoma. Bone Marrow Transplant. 2021; 56(1): 155–66. (3iiiA)

18)Fuji S, et al. A decision analysis comparing unrelated bone marrow transplantation and cord blood transplantation in patients with aggressive adult T-cell leukemia-lymphoma. Int J Hematol. 2020; 111(3): 427–33. (3iiiA)

19)Tamaki H, et al. Donor-derived T-cell leukemia after bone marrow transplantation. N Engl J Med. 2006; 354(16): 1758–9.

20)Eligibility Criteria for Related-donor Hematopoietic Stem Cell Donor (Bone Marrow/peripheral Blood) Enrollment in Donor Accident Insurance (August 2015, Version 2.2), The Japan Society for Hematopoietic Cell Transplantation Donor Committee.

21)Fuji S, et al. Pretransplantation Anti-CCR4 Antibody Mogamulizumab Against Adult T-Cell Leukemia/Lymphoma Is Associated With Significantly Increased Risks of Severe and Corticosteroid-Refractory Graft-Versus-Host Disease, Nonrelapse Mortality, and Overall Mortality. J Clin Oncol. 2016; 34(28): 3426–33. (3iiiA)


**CQ4 Is use of cord blood or an HLA-haploidentical donor useful in allogeneic HSCT for aggressive ATL?**

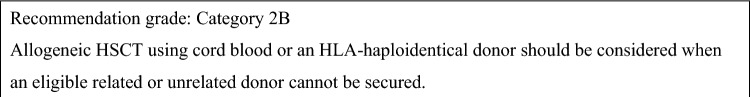



## Explanation

Allogeneic HSCT is considered in patients with aggressive ATL who respond to treatment, with a related donor or unrelated donor from Marrow Donor Program being the first choice. However, in some cases patients become refractory to treatment before they can undergo allogeneic HSCT due to factors such as advanced age of potential related donors and the time required to coordinate marrow donors. Cord blood transplantation and HLA-haploidentical transplantation have been explored as a way to address this issue, and several retrospective analyses on these options have been published. A retrospective analysis of a large database including data from the late 1990s to the early 2000s showed that cord blood transplantation not only had a 3-year OS rate of 17%, which was inferior to both related and unrelated transplantation, but also had a higher treatment-related mortality rate.^1^ Subsequent studies showed that good disease status at the time of transplantation improves outcomes.^2–6^ Another study showed that GVHD-free, relapse-free survival rates do not differ from those observed with related or unrelated donors.^7^ Moreover, a prospective cohort analysis examining recent trends in transplantation reported that 2-year OS rate and non-relapse mortality for cord blood and HLA-haploidentical transplantation did not differ significantly from figures for transplantation from a related or unrelated donor.^8^ A phase I/II trial of HLA-haploidentical transplantation with cyclophosphamide for GVHD prophylaxis demonstrated its tolerability and efficacy.^9^

In summary, although an HLA-matched related or unrelated donor is the first choice, use of cord blood or an HLA-haploidentical donor should be considered when an eligible HLA-matched related or unrelated donor cannot be secured.

## References

1)Hishizawa M, et al. Transplantation of allogeneic hematopoietic stem cells for adult T-cell leukemia: a nationwide retrospective study. Blood. 2010; 116(8): 1369–76. (3iiiA)

2)Fukushima T, et al. Feasibility of cord blood transplantation in chemosensitive adult T-cell leukemia/ lymphoma: a retrospective analysis of the Nagasaki Transplantation Network. Int J Hematol. 2013; 97(4): 485–90. (3iiiA)

3)Kato K, et al. Treatment of patients with adult T-cell leukemia/lymphoma with cord blood transplantation: a Japanese nationwide retrospective survey. Biol Blood Marrow Transplant. 2014; 20(12): 1968–74. (3iiiA)

4)Nakamura T, et al. Unrelated cord blood transplantation for patients with adult T-cell leukemia/lymphoma: experience at a single institute. Int J Hematol. 2012; 96(5): 657–63. (3iiiA)

5)Nakano N, et al. Cord blood transplantation with a reduced-intensity conditioning regimen using fludarabine and melphalan for adult T-cell leukemia/lymphoma. Int J Hematol. 2021; 113(6): 861–71. (3iiiA)

6)Yoshimitsu M, et al. A retrospective analysis of haplo-identical HLA-mismatch hematopoietic transplantation without posttransplantation cyclophosphamide for GVHD prophylaxis in patients with adult T-cell leukemia-lymphoma. Bone Marrow Transplant. 2019; 54(8): 1266–74. (3iiiA)

7)Muranushi H, et al. GVHD-free, relapse-free survival provides novel clues for optimizing allogeneic-HSCT for adult T-cell leukemia/lymphoma. Bone Marrow Transplant. 2021; 56(1): 155–66. (3iiiA)

8)Ito A, et al. Improved survival of patients with aggressive ATL by increased use of allo-HCT: a prospective observational study. Blood Adv. 2021; 5(20): 4156–66. (3iiiA)

9)Tanaka T, et al. A Phase I/II Multicenter Trial of HLA-Haploidentical PBSCT with PTCy for Aggressive Adult T Cell Leukemia/Lymphoma. Transplant Cell Ther. 2021; 27(11): 928.e1–7. (3iiiDiv)


**CQ5 What treatments are recommended for relapsed or refractory ATL?**

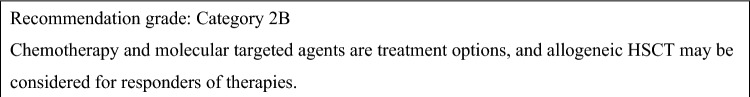



## Explanation

Various regimens for relapsed/refractory aggressive ATL have been developed to date. All studies have shown transient responses, but response duration is short. Small phase II trials of EPOCH, sobuzoxane (MST-16), and irinotecan were conducted in Japan. The overall response rate (ORR) ranges 38–57%, but response was only maintained for 1–6 months.^1–3^ Since then, several new drugs have been investigated in clinical trials from 2012 onward. The anti-CCR4 antibody mogamulizumab had an ORR of 31% in a phase I trial, and an ORR of 50% and median OS of 13.7 months in a phase II trial.^4,5^ A long-term follow-up analysis showed that 31% of patients in the phase I trial survived for 3 years or longer, and the 3-year OS rate in the phase II trial was 23%.^6^ The Lenalidomide, immunomodulatory drug, had an ORR of 33% in a phase I trial, and ORR of 42% and median OS of 20.3 months in a phase II trial.^7,8^ Tucidinostat, an oral histone deacetylase inhibitor, had a 30% ORR and a median OS of 7.9 months in a phase II trial in patients with relapsed or refractory ATL previously treated with mogamulizumab.^9^ Valemetostat, an inhibitor of the histone methyltransferases EZH1 and EZH2, had an ORR of 48% and median OS of 16.4 months in a phase II trial in patients with relapsed or refractory ATL previously treated with mogamulizumab.^10^ These four drugs are currently (as of June 2023) covered by the NHI in Japan for the treatment of relapsed or refractory ATL (mogamulizumab is also covered by the NHI for previously untreated ATL). Brentuximab vedotin, an anti-CD30 antibody‒drug conjugate, demonstrated efficacy in a phase II trial in CD30-positive relapsed or refractory PTCL, and is now available for CD30-positive relapsed or refractory ATL.^11^ Several studies have shown that patients with aggressive ATL relapsed on or refractory to chemotherapy can achieve long-term survival if they respond to treatment.^12,13^ ATL relapsed after allogeneic HSCT has a very poor prognosis, but donor lymphocyte infusion may be effective.^14^ Radiotherapy is considered for local control and symptom palliation, as studies have shown that radiotherapy can successfully control local ATL-related lesions that are refractory to chemotherapy.^15,16^

## References

1)Toriyama E, et al. EPOCH regimen as salvage therapy for adult T-cell leukemia-lymphoma. Int J Hematol. 2018; 108(2): 167–75. (3iiiA)

2)Ohno R, et al. Treatment of adult T-cell leukemia/lymphoma with MST-16, a new oral antitumor drug and a derivative of bis (2,6-dioxopiperazine). The MST-16 Study Group. Cancer. 1993; 71(7): 2217–21. (3iiiDiv)

3)Tsuda H, et al. Treatment of adult T-cell leukaemia-lymphoma with irinotecan hydrochloride (CPT-11). CPT-11 Study Group on Hematological Malignancy. Br J Cancer. 1994; 70(4): 771–4. (3iiiDiv)

4)Yamamoto K, et al. Phase I study of KW-0761, a defucosylated humanized anti-CCR4 antibody, in relapsed patients with adult T-cell leukemia-lymphoma and peripheral T-cell lymphoma. J Clin Oncol. 2010; 28(9): 1591–8. (3iiiDiv)

5)Ishida T, et al. Defucosylated anti-CCR4 monoclonal antibody (KW-0761) for relapsed adult T-cell leukemia-lymphoma: a multicenter phase II study. J Clin Oncol. 2012; 30(8): 837–42. (3iiiDiv)

6)Ishida T, et al. Mogamulizumab for relapsed adult T-cell leukemia-lymphoma: Updated follow-up analysis of phase I and II studies. Cancer Sci. 2017; 108(10): 2022–9. (3iiiDiv)

7)Ogura M, et al. Lenalidomide in relapsed adult T-cell leukaemia-lymphoma or peripheral T-cell lymphoma (ATLL-001): a phase 1, multicentre, dose-escalation study. Lancet Haematol. 2016; 3(3): e107–18 (3iiiDiv)

8)Ishida T, et al. Multicenter Phase II Study of Lenalidomide in Relapsed or Recurrent Adult T-cell Leukemia/Lymphoma: ATLL-002. J Clin Oncol. 2016; 34(34): 4086–93. (3iiiDiv)

9)Utsunomiya A, et al. Oral histone deacetylase inhibitor tucidinostat (HBI-8000) in patients with relapsed or refractory adult T-cell leukemia/lymphoma: Phase IIb results. Cancer Sci. 2022; 113(8): 2778–87. (3iiiDiv)

10)Izutsu K, et al. An Open-Level, Single-Arm, Phase 2 trail of Valemetostat in Relapsed or Refractory Adult T-cell Leukemia/Lymphoma. Blood. 2023; 141(9): 1159–68. (3iiiDiv)

11)Horwitz S, et al. Brentuximab vedotin with chemotherapy for CD30-positive peripheral T-cell lymphoma (ECHELON-2): a global, double-blind, randomised, phase 3 trial. Lancet. 2019; 393(10168): 229–40. (1iDiii)

12)Fukushima T, et al. Allogeneic hematopoietic stem cell transplantation provides sustained long-term survival for patients with adult T-cell leukemia/lymphoma. Leukemia. 2005; 19(5): 829–34. (3iiiA)

13)Kato K, et al. Allogeneic bone marrow transplantation from unrelated human T-cell leukemia virus-I-negative donors for adult T-cell leukemia/lymphoma: retrospective analysis of data from the Japan Marrow Donor Program. Biol Blood Marrow Transplant. 2007; 13(1): 90–9. (3iiiA)

14)Kato K, et al. The outcome and characteristics of patients with relapsed adult T cell leukemia/lymphoma after allogeneic hematopoietic stem cell transplantation. Hematol Oncol. 2019; 37(1): 54–61. (3iiiA)

15)Simone CB 2nd, et al. Radiation therapy for the management of patients with HTLV-1-associated adult T-cell leukemia/lymphoma. Blood. 2012; 120(9): 1816–9. (3iiiDiv)

16)Maemoto H, et al. Appropriate radiation dose for symptomatic relief and local control in patients with adult T cell leukemia/lymphoma. J Radiat Res. 2019; 60(1): 98–108. (3iiiDiv)


**CQ6 Is early therapeutic intervention beneficial for indolent ATL?**

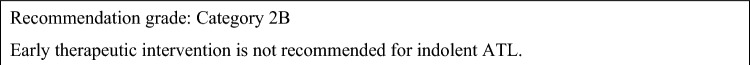



## Explanation

In a retrospective analysis of 337 patients with smoldering or chronic ATL at 40 institutions in the Kyushu region and Okinawa in Japan,^1^ MST was 5.2 years for smoldering ATL and 3.6 years for chronic ATL. A subgroup analysis showed no difference in OS between no treatment and anticancer drug therapy for smoldering ATL, but significantly longer OS with no treatment for chronic ATL (MST 7.4 years vs. 2.0 years). In a multicenter study conducted in the Kyushu region of Japan between 1988 and 1997, MST in the 26 patients diagnosed with smoldering ATL was 7.3 years (median observation time 6.5 years).^2^ In a single-center retrospective study in which a total of 90 patients diagnosed with smoldering ATL (*n* = 25) or chronic ATL (37 with unfavorable prognostic factors, 26 without unfavorable prognostic factors, and 2 with unknown unfavorable prognostic factor status) between 1974 and 2003 were observed without treatment until disease progression, median observation time was 4.1 years and 12 patients survived for 10 years or longer. However, the long-term prognosis of these patients was poor as illustrated by the 2-, 5-, 10-, and 15-year OS rates of approximately 60%, 47%, 23%, and 13%, respectively.^3^ MST and progression-free MST were 4.1 years and 3.3 years, respectively. Therefore, MST after disease progression is estimated to be approximately 1 year, and although a certain percentage of patients achieve long-term survival, the prognosis after disease progression is poor.

In a retrospective analysis of 248 patients with indolent ATL (smoldering or chronic) diagnosed between 2000 and 2009, patients were classified as high-, intermediate- or low-risk based on sIL2R as a prognostic factor. MST for these risk groups was 1.6 years, 5.5 years, and not reached, respectively, indicating that the high-risk group has a poor prognosis.^4^

In summary, the long-term prognosis of indolent ATL is by no means good. However, the Japanese medical community has reached the consensus that a watch-and-wait until progression to aggressive ATL is the best approach due to the lack of effective treatments for indolent ATL.

It is recommended to reference the Guidelines for the Management of Cutaneous Malignancies with regard to skin-directed therapy for patients with smoldering ATL only presenting with skin lesions,^5^ as well as the manual explaining how hematologists and dermatologists should use the guidelines when collaborating in the treatment of an ATL patient with skin lesions.^6^ Most indolent ATL patients with skin lesions survive for a long time, but there have been many reports of certain characteristics such as tumoral lesions being associated with a poor prognosis in indolent ATL. Though an extranodal primary cutaneous category of lymphoma ATL has been proposed, aspects such as definitions of skin lesion characteristics have not been established.^7^
